# LXRα Regulates oxLDL-Induced Trained Immunity in Macrophages

**DOI:** 10.3390/ijms23116166

**Published:** 2022-05-31

**Authors:** Hannes M. Findeisen, Vivienne C. Voges, Laura C. Braun, Jannik Sonnenberg, Dennis Schwarz, Helena Körner, Holger Reinecke, Yahya Sohrabi

**Affiliations:** Department of Cardiology I—Coronary and Peripheral Vascular Disease, Heart Failure, University Hospital Münster, 48149 Münster, Germany; vivienne.voges@ukmuenster.de (V.C.V.); laura.braun@ukmuenster.de (L.C.B.); Jannik.Sonnenberg@ukmuenster.de (J.S.); Dennis.Schwarz@ukmuenster.de (D.S.); helena.koerner@ukmuenster.de (H.K.); holger.reinecke@ukmuenster.de (H.R.)

**Keywords:** LXR, trained innate immunity, oxLDL, immunometabolism, inflammation

## Abstract

Reprogramming of metabolic pathways in monocytes and macrophages can induce a proatherosclerotic inflammatory memory called trained innate immunity. Here, we have analyzed the role of the Liver X receptor (LXR), a crucial regulator of metabolism and inflammation, in oxidized low-density lipoprotein (oxLDL)-induced trained innate immunity. Human monocytes were incubated with LXR agonists, antagonists, and oxLDL for 24 h. After five days of resting time, cells were restimulated with the TLR-2 agonist Pam3cys. OxLDL priming induced the expression of *LXRα* but not *LXRβ*. Pharmacologic LXR activation was enhanced, while LXR inhibition prevented the oxLDL-induced inflammatory response. Furthermore, LXR inhibition blocked the metabolic changes necessary for epigenetic reprogramming associated with trained immunity. In fact, enrichment of activating histone marks at the *IL-6* and *TNFα* promotor was reduced following LXR inhibition. Based on the differential expression of the LXR isoforms, we inhibited *LXRα* and *LXRβ* genes using siRNA in THP1 cells. As expected, siRNA-mediated knock-down of *LXRα* blocked the oxLDL-induced inflammatory response, while knock-down of *LXRβ* had no effect. We demonstrate a specific and novel role of the LXRα isoform in the regulation of oxLDL-induced trained immunity. Our data reveal important aspects of LXR signaling in innate immunity with relevance to atherosclerosis formation.

## 1. Introduction

Today, atherosclerosis can be best described as a lipid-driven low-grade chronic inflammatory disease of the vascular wall [1,2]. The accumulation of lipoproteins, mainly low-density lipoproteins (LDL) in the subendothelial space fostered by endothelial dysfunction, is generally considered the initial step during the pathogenesis of atherosclerosis [2]. In the intimal layer, phagocytic cells such as macrophages or transformed smooth muscle cells take up cholesterol complexes [2,3]. Intramural lipoproteins can be modified through interactions with metal ions, reactive oxygen species (ROS), and enzymes, such as myeloperoxidase [4]. These modified lipoproteins, for example, oxidized LDL (oxLDL), can serve as so-called damage-associated molecular pattern molecules (DAMPs), which can activate pattern recognition receptors (PRR), the central regulators of the immune response. Lipid-mediated activation of PRRs, such as toll-like receptors (TLRs), triggers proinflammatory signaling pathways, thereby inducing the secretion of various chemokines or cytokines such as IL-1β, IL-6, or TNFα [2]. These inflammatory mediators further activate immune cells, smooth muscle cells and endothelial cells resulting in a chronic inflammatory response [2,5,6]. Additional studies have demonstrated that DAMP activation through oxLDL can induce a trained innate immunity in human monocytes [5,6,7,8,9]. Trained innate immunity describes a lasting activation or memory-like state that enhances the immune response of innate immune cells and even non-immune cells to a secondary challenge [10,11]. While the trained innate immunity phenotype can confer protection from infectious diseases, activation by non-microbial atherogenic triggers such as oxLDL or Lipoprotein (a) can have harmful consequences [10,12,13]. Current evidence has consistently implicated metabolic and epigenetic mechanisms in regulating trained innate immunity [10,12,14]. Training of monocytes was associated with a shift from oxidative phosphorylation to glycolysis resulting in increased lactate production in an Akt/mTOR/Hif1α-dependent manner [5,7,8]. Furthermore, activation of fatty acid synthesis and cholesterol metabolism have been demonstrated to control trained immunity [14,15]. Downstream, metabolic reprogramming controls the modulation of the epigenetic landscape by implementing a sustained pattern of activation. Recently, we have shown that Liver X receptors (LXRs), key regulators of cholesterol and fatty acid metabolism, are involved in the regulation of trained innate immunity [16]. Using an established in vitro model of trained innate immunity in human monocytes, we showed that inhibition of LXR blocks Bacillus Calmette-Guerin vaccine (BCG)-induced trained immunity [16]. Furthermore, LXR agonist treatment induced a long-term inflammatory response in monocytes independent of any additional stimuli [16]. This inflammatory stimulation by LXR agonists was accompanied by characteristic features of trained innate immunity, such as activating histone marks on inflammatory gene promoters and metabolic reprogramming with increased HIF1α-signaling [16,17]. The role of LXR activation in the development of innate immune memory has not been addressed, therefore, we have investigated the role of LXR in oxLDL-induced trained innate immunity in this study.

## 2. Results

### 2.1. LXR Agonists Augment, Whereas LXR Inhibitors Block oxLDL-Induced Inflammatory Innate Immune Memory

**Figure 1:**
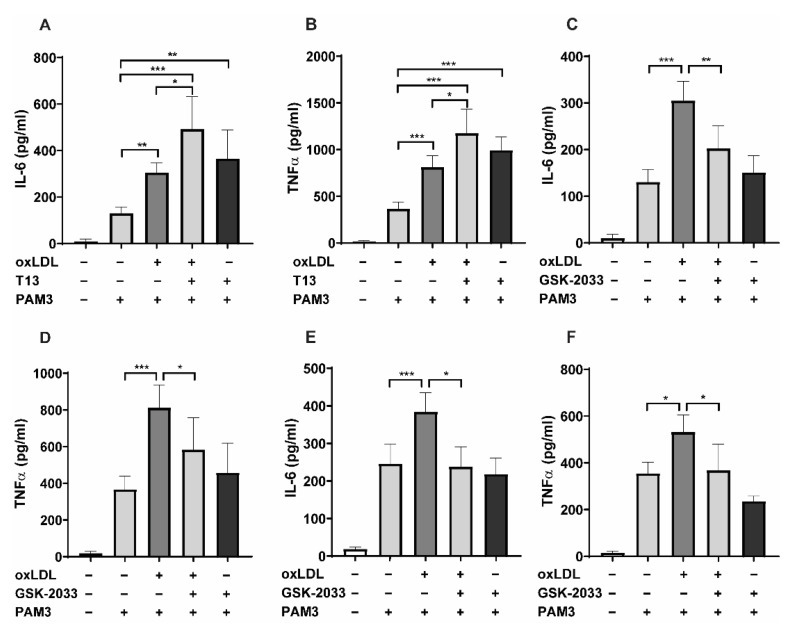
Liver X receptor (LXR) dependent regulation of the oxidized low-density lipoprotein (oxLDL)-induced proinflammatory phenotype in human monocytes. Monocytes were treated as indicated with 20 μg/mL oxLDL, 2 μM T1317 (LXR agonist), and 2.5 μM GSK2033 (LXR antagonist) or vehicle for 24 h, kept for 5 days in a complete medium, and restimulated with 5 μg/mL Pam3cys for 24 h. IL-6 (**A**,**C**) and TNFα (**B**,**D**) were measured in the supernatant. THP1 cells were differentiated to macrophages and were primed as described above; the cells were rested for 5 days and restimulated with 10 μg/mL Pam3cys for 24 h. IL-6 (**E**) and TNFα (**F**) were measured in the supernatant. Graphs represent mean values ± SD of six individuals in three different experiments. * *p* < 0.05, ** *p* < 0.01, and *** *p* < 0.001.

We have previously shown that pharmacologic activation of LXR during BCG treatment (a well-studied inducer of trained innate immunity) can strongly enhance inflammatory cytokine synthesis upon restimulation with the TLR-2 agonist Pam3cys, whereas BCG-priming was inhibited following inhibition of LXR [16]. Based on this observation, we have analyzed the role of LXR in oxLDL-induced trained innate immunity. Human monocytes were pretreated with the LXR agonist (T1317) and trained with low-dose oxLDL for 24 h. Following a 5-day resting period, cells were restimulated with Pam3cys. Initially, the oxLDL effect in inducing innate immune memory was tested with 10 and 20 µg/mL. However, the experiments were continued using 20 µg/mL oxLDL due to stronger inflammatory responses in comparison to 10µg/mL. In accordance with our previous BCG experiment [16], LXR activation enhanced IL-6 and TNFα secretion in oxLDL-primed cells (Figure 1A,B), while inhibition of LXR with the antagonist GSK2033 reduced the priming effect of oxLDL-training (Figure 1C,D) after Pam3cys restimulation. As was shown before, LXR activation using LXR agonist alone enhanced inflammatory responses to restimulation [16,17]. Without restimulation, the primed cells did not produce detectable cytokine levels (data not shown). Furthermore, treatment of monocytes with the LXR inverse agonist (SR9238) significantly reduced the levels of proinflammatory cytokines as well (Figure S1A,B). In addition, we developed a model of oxLDL-induced trained innate immunity in the human monocytic cell line THP-1. Similar to human primary monocytes, GSK2033 blocked oxLDL-training in THP-1 cells (Figure 1E,F). Neither PMA treatment, which induces differentiation, nor oxLDL treatment of PMA-differentiated THP-1 cells resulted in a measurable response of inflammatory cytokines without TLR restimulation.

### 2.2. LXR Inhibition Alters Metabolic Reprogramming

**Figure 2:**
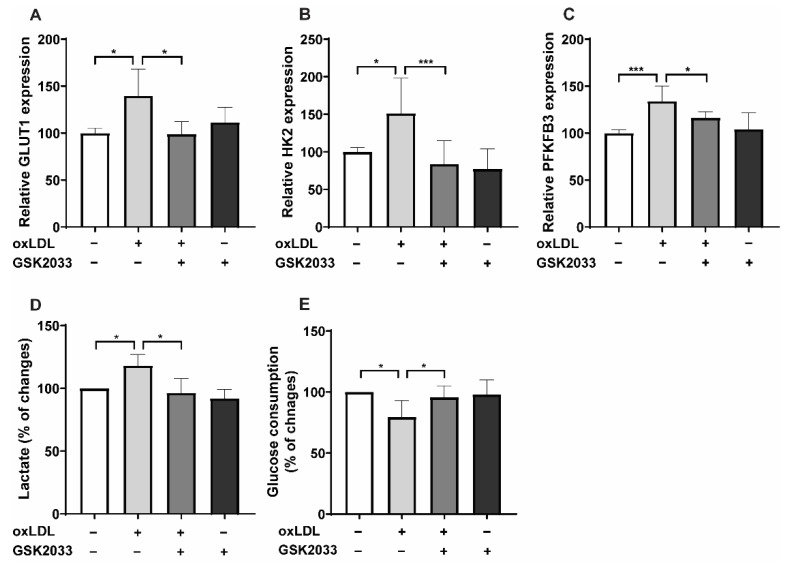
LXR inhibition alters oxLDL-induced metabolic reprogramming. Monocytes were treated as indicated with 20 μg/mL oxLDL, 2.5 μM GSK2033 (LXR antagonist), or vehicle for 24 h and either lysed for RNA expression or kept for 5 days in complete medium for lactate assays and glucose consumption. mRNA levels of *GLUT1*, *HK2,* and *PFKFB3* were analyzed by real-time qPCR (**A**–**C**). On day 6 lactate concentration was measured in the cell lysate and glucose concentration was measured in the supernatant (**D**,**E**). Graphs represent mean values ± SD of six individuals in three different experiments. * *p* < 0.05 and *** *p* < 0.001.

The metabolic shift toward aerobic glycolysis is a crucial mechanism in establishing trained innate immunity [14]. This can be seen with many different inducers of trained immunity, including oxLDL [18]. Enhanced aerobic glycolysis during trained immunity is associated WITH and regulated through increased expression of key glycolytic enzymes such as *GLUT1*, *HK2*, and *PFKFB3*. Likewise, increased gene expression was blocked following LXR inhibition (Figure 2A–C), which was confirmed by *PFKFB3* protein analysis by Western blotting (Figure S4A–C). Accordingly, we found a shift toward glycolysis characterized by higher lactate production and glucose consumption (Figure 2D,E). Interestingly, the oxLDL-induced metabolic changes in lactate and glucose consumption were reversed following LXR-inhibitor treatment (Figure 2D,E). In addition, LXR inverse agonist SR9238 inhibited the oxLDL-induced expression of glycolytic genes 24 h after priming (Figure S2A–C).

### 2.3. LXR Inhibition Prevents Epigenetic Modifications in Human Monocytes

**Figure 3:**
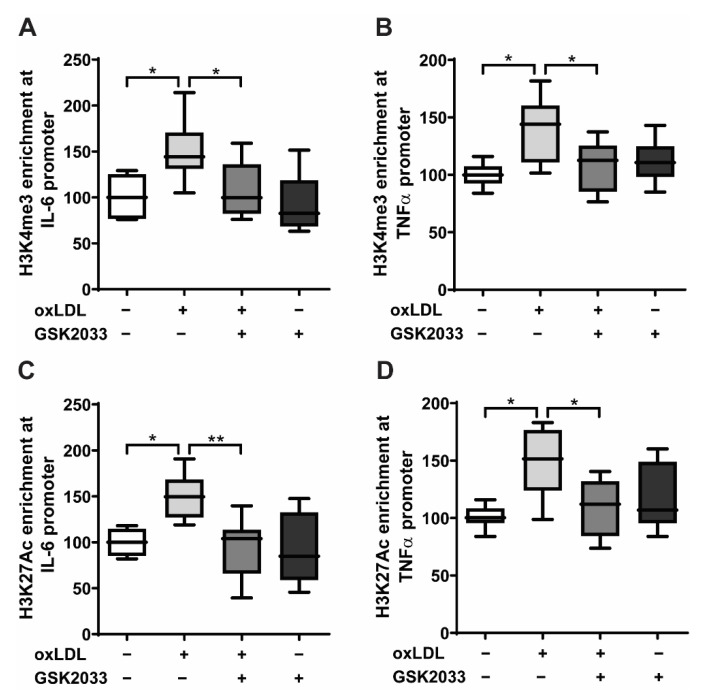
LXR inhibition inhibits epigenetic reprogramming. Monocytes were treated as indicated with 20 μg/mL oxLDL, 2.5 μM GSK2033 (LXR antagonist), or vehicle for 24 h and kept for 5 days in a complete medium. Chromatin was collected and a ChIP-PCR assay was performed using an antibody against histone H3 acetylated at lysine 27 (H3K27ac), histone H3 trimethylated at lysine 3 (H3K4me3), or control IgG. Then, quantitative real-time PCR was performed with primers for *IL-6* (**A**,**C**) and *TNFα* (**B**,**D**). Graphs represent mean values ± SD of six individuals in three different experiments. * *p* < 0.05 and ** *p* < 0.01.

Metabolic reprogramming implements lasting epigenetic modifications of histone proteins such as acetylation of H3 lysine 27 (H3K27ac) or histone H3 lysine 4 trimethylation (H3K4me3) on the promoters of inflammatory genes—a process that is essential for the development of an innate immune memory [10,16]. Therefore, we investigated whether LXR inhibition blocks oxLDL-induced epigenetic reprogramming. As demonstrated in Figure 3A–D, oxLDL priming resulted in a significant increase of the histone modifications H3K27ac and H3Kme3 on the promoter of *IL-6* and *TNFα*. In contrast, LXR antagonist treatment inhibited oxLDL-induced histone modifications.

### 2.4. LXR Inhibition Alters oxLDL-Induced Pathways by Downregulating LXR Target Gene Expression

**Figure 4:**
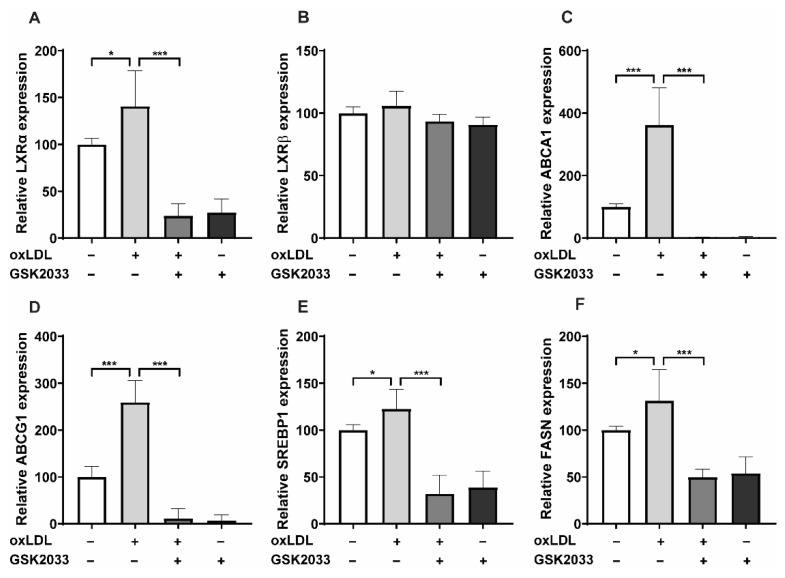
GSK2033 inhibits oxLDL induced upregulation of LXR target genes. Monocytes were treated as indicated with 20 μg/mL oxLDL, 2.5 μM GSK2033 (LXR antagonist), or vehicle for 24 h and lysed for RNA expression. mRNA levels of *LXRα*, *LXRβ*, *ABCA1*, *ABCG1*, *SREBP1* and *FASN* were analyzed by real-time qPCR (**A**–**F**). Graphs represent mean values ± SD of six individuals in three different experiments. * *p* < 0.05 and *** *p* < 0.001.

OxLDL priming selectively increased the expression of the *LXRα*-isoform and downstream LXR target genes involved in cholesterol efflux (*ABCA1*, *ABCG1*) as well as fatty acid synthesis (*SREBP1, FASN*) in human monocytes and THP-1 cells (Figure 4A–F and Figure S3A–C). As expected, LXR inhibition with GSK2033 or SR9238 prevented the induction of *LXRα*, *ABCA1*, *ABCG1*, *SREBP1,* and *FASN* (Figure 4A–F, Figures S3A–F and S4A–B). We have previously shown that Acetyl-CoA levels, the substrate of fatty acid synthesis, are increased by LXR agonist treatment in human monocytes [16]. Furthermore, adding Acetyl-CoA to human monocytes induced some degree of inflammatory response itself [16]. Apart from *SREBP1* and *FASN* (the regulators of fatty acid synthesis), oxLDL-priming elevates Acetyl-CoA levels and induces the expression of *ACLY*, which plays a vital role in Acetyl-CoA synthesis. Again, GSK-2033 treatment inhibited increased *ACLY* expression levels and reduced Acetyl-CoA levels (Figure S5A,B).

### 2.5. LXRα Is the Primary Regulator of oxLDL-Induced Trained Immunity, Not LXRβ

As demonstrated in Figure 4A,B, oxLDL treatment selectively induced the α-isoform of LXR, while LXRβ remained unchanged. In order to investigate whether the inhibition of LXRα is sufficient to stop oxLDL training, we performed siRNA experiments in THP-1 cells, which provide more stable and reproducible experimental conditions than primary monocytes (Figure S6A–C).

**Figure 5:**
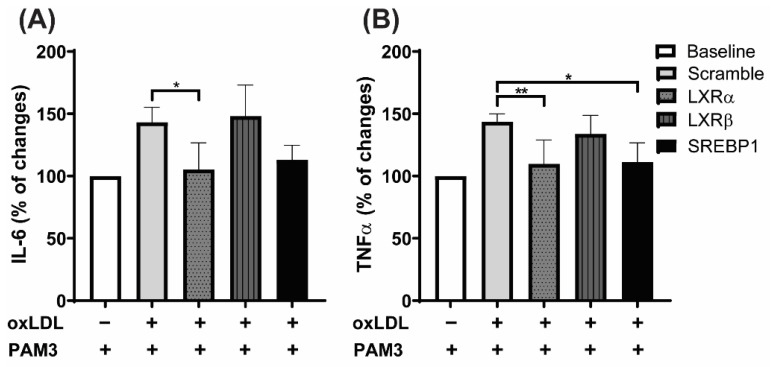
LXRα and SREBP1 play an important role in regulating oxLDL-induced trained immunity. Differentiated THP1 cells were transfected with siRNA against *LXRα*, *LXRβ,* and *SREBP1* or scrambled siRNA and treated with 20 μg/mL oxLDL or vehicle for 24 h. Cells were rested for 5 days and restimulated with 10 μg/mL Pam3cys for 24 h. IL-6 and TNFα were measured in the supernatant (**A**,**B**). Graphs represent mean values ± SD of three different experiments. * *p* < 0.05 and ** *p* < 0.01.

Confirming a specific role for LXRα, siRNA-mediated knock-down of *LXRα* but not *LXRβ* diminished inflammatory cytokine production in oxLDL treated cells upon Pam3cys restimulation (Figure 5A,B). Interestingly, knocking down *SREBP1* reduced the levels of inflammatory cytokines (Figure 5A,B), indicating a pivotal role for lipid synthesis in oxLDL priming.

## 3. Discussion

Deposition and modifications of lipids and lipoproteins in the vascular wall constitute the main trigger for atherosclerosis formation. The interactions between these molecules and immune cells activate complex signaling networks resulting in chronic inflammation. Recent studies have demonstrated the involvement of trained innate immunity in the regulation of this chronic vascular inflammation. Initially, Bekkering et al. have shown that exposure of human monocytes for 24 h to oxLDL results in increased expression of the inflammatory mediators *TNFα*, *IL-6*, *MCP-1,* and *MMP-9* as well as increased foam cell formation upon restimulation with TLR-2 and -4 agonists 6 days after initial priming [8]. Additional studies have begun to unravel the mechanisms involved in oxLDL-induced trained immunity. As in other models of trained immunity, upregulation of aerobic glycolysis appears to be a central component of the associated metabolic reprogramming [18]. Increased oxidative phosphorylation and production of ROS are prerequisites for oxLDL-induced trained immunity [7,19]. Downstream of glycolysis and the TCA-cycle, which provides Acetyl-CoA for subsequent metabolic pathways, fatty acid metabolism and cholesterol synthesis participate in trained innate immunity [19]. This is highlighted by the observation that statin treatment, which blocks HMG-CoA reductase (the key enzyme of the mevalonate pathway) inhibits oxLDL-induced trained immunity [14,15]. Conversely, adding mevalonate, which is synthesized downstream of HMG-CoA reductase, rescued the statin effect [15]. The contribution of trained immunity to atherosclerosis or atherosclerotic phenotypes has been demonstrated in vivo and ex vivo. Transient exposure of LDL-receptor knockout mice to an atherogenic Western diet resulted in a sustained epigenetic reprogramming of myeloid cells with an enhanced propensity for inflammasome activation following restimulation [20]. Subsequently, lipid synthesis plays a crucial role in IL-1β secretion, which is a key inducer of innate immune memory [21]. Human monocytes isolated from patients with known atherosclerosis display a pro-inflammatory phenotype with increased glycolysis and epigenetic reprogramming [9]. When considering the fundamental role of lipid metabolism in trained immunity, a significant role for LXR-signaling seems likely. To our knowledge, this is the first study to look at LXR-signaling in regulating oxLDL-induced trained immunity in vitro, however, further studies are warranted to confirm the data in vivo or ex vivo. We have previously shown that pharmacologic activation of LXR induces a trained immunity phenotype in human monocytes independent of additional stimuli [16]. Similar to conventional triggers of trained immunity, LXR activation induced aerobic glycolysis, Acetyl-CoA, and Cholesterol-synthesis. In mice, accumulating evidence has shown that LXR activation exerts anti-inflammatory actions that result in the modulation of immune responses [22,23,24]. In contrast to mice, experiments in human cells demonstrated proinflammatory effects of LXR agonists [17,25,26]. The mechanisms through which LXR regulates glycolysis or the transcription of glycolytic genes in human monocytes remain largely elusive. However, using a different macrophage cell culture model recently, Menegaut et al. demonstrate that LXR activation induces LXR-recruitment to the HIF1α-promoter as well as to HIF1α binding sites on the promoter of glycolytic genes with increased transcription and activation of HIF1α-dependent glycolysis [17,27,28]. In line with our observations, GLUT1 and HK2 were among the regulators of glucose metabolism that were activated through the LXR-HIF1α-pathway [17,28]. Interestingly, this role of LXR in cellular glucose metabolism appears to be specific to human macrophages compared to murine cells [17,29]. This observation is crucial as the interplay of LXR-signaling and inflammation has been extensively studied in mice and murine cells, resulting in the establishment of a model with a predominantly anti-inflammatory role for LXR. Previous studies have described a more complex role for LXR in inflammation and immunity in human cells, independently of our model of trained immunity. Fontaine et al. have demonstrated that pretreatment of human macrophages with LXR agonists resulted in a significantly increased response to LPS stimulation through transcriptional upregulation of TLR-4 [26]. Likewise, the observation was confirmed to be absent in murine macrophages [17]. Similarly, treatment of human dendritic cells with LXR agonists enhanced the expression of inflammatory cytokines following stimulation with TLR-3 or -4 agonists [30]. LXR activation has been shown to directly regulate *IL-1β* expression, a master regulator of inflammation in general and trained immunity in particular [16,17,31]. Likewise, GSK2033 (LXR antagonist) treatment inhibited LXR, ABCA1, HIF1α, and IL-1β upregulation in monocytes treated with conditional medium containing plaques homogenates [17]. Data on the role of LXR signaling in lipid metabolism and inflammation in human cells are largely lacking. A recent paper by Liebergall et al. has described a novel role for LXR-controlled lipid metabolism in TLR-induced inflammation [32]. Stimulation of mouse macrophages and human THP-1 cells induced LXRα expression in an Interferon-β-dependent manner. Activation of LXR together with mTOR- and SREBP1c-signaling regulated an increase in fatty acid and cholesterol biosynthesis at later stages of the TLR response [32]. LXR activation did not increase cholesterol efflux or upregulate related genes. The authors concluded that increased synthesis of lipid mediators was necessary for resolving the inflammatory response [32]. The missing induction of LXR target genes involved in cholesterol efflux distinguishes oxLDL stimulation from cell culture models using specific TLR-agonists, although both treatments activate TLR-signaling [32,33]. In fact, oxLDL is a well-known ligand to the scavenger receptor CD36 which in turn can activate TLR-2 or -4 signaling. This interaction of CD36 and TLR-signaling in response to oxLDL is a prime example of DAMP-induced sterile inflammation and a prerequisite for the induction of trained immunity [34,35]. Notably, TLR activation induced LXRα expression, while LXRβ remained unchanged [32], which is in line with our observation in oxLDL-treated cells. In general, tissue distribution of the LXR isoforms varies. LXRα is primarily expressed in macrophages, intestine, liver, adipose, and kidney, whereas LXRβ expression is more ubiquitous [36]. Previous studies have already demonstrated a specific role for LXRα in inflammation and atherosclerosis. Chawla et al. demonstrated a LXRα specific regulation of LXR-dependent cholesterol efflux through PPARγ in THP-1 cells [37]. The lack of PPARγ increased atherosclerosis formation in a mouse model. Additionally, Castrillo et al. demonstrated the inhibition of anti-inflammatory LXR-signaling following TLR activation in murine cells with increased LXRα expression and unchanged expression of LXRβ in response to bacterial infection [38]. Therefore, a specific role for LXRα in inflammation and trained immunity is conceivable. However, as mentioned above, there is ample evidence that the role of LXR in the regulation of inflammation varies considerably between humans and mice. Consequently, we think that all mouse data on LXR in immune cells have only limited relevance for human cells and our cell culture model. Additional research is necessary to analyze the role of LXR isoforms regarding their effect on glycolysis, inflammation, and trained immunity.

## 4. Materials and Methods

### 4.1. Monocyte Isolation and Culture

Human monocytes were isolated from healthy donors as described earlier [7,16]. The fresh human blood leucocyte reduction chambers of plate apheresis sets were collected from the blood bank of the University Hospital Münster in accordance with the Declaration of Helsinki on Ethical Principles for Medical Research (anonymous healthy donors aged 18–55 years, no personal information was shared). Monocyte isolation was performed by differential density centrifugation over Histopaque^®^ 1077 (Sigma-Aldrich, St Louis, MO, USA, #10771) using Leucosep^®^ tubes (50 mL, with filter, Greiner, Frickenhausen, Germany, #227290) for 20 min at 615× *g* with no brake. The cells were resuspended in PBS and centrifuged at RT, one time at 550× *g* for 10 min, then three times at 350× *g* for 8 min. In order to enrich monocytes, a second density centrifugation using a Percoll gradient was performed. Briefly, 150–200 × 10^6^ PBMCs were resuspended in RPMI-1640 (Sigma, Paisley, UK, #R8758) with 10% FBS (Sigma, Sigma, St Louis, MO, USA #F7524), layered on top of a hyper-osmotic Percoll (GE Healthcare, Uppsala, Sweden, #17089101) solution (46% Percoll and PBS, 10% FBS RPMI) and centrifuged for 30 min at 580× *g*. The interphase layer was isolated and cells were washed with PBS. Cells were purified further with MACS Pan Monocyte Isolation Kit (Miltenyi Biotec, Bergisch Gladbach, Germany, #130-096-537) and washed once with serum-free RPMI-1640 medium before resuspension in RPMI culture medium supplemented with 10% pooled AB human serum (Sigma, St Louis, MO, USA, #H4522), 1% penicillin/streptomycin (Gibco, Paisley, UK, #15140122), and 5 mM glucose (Sigma, St Louis, MO, USA, #G8644).

### 4.2. LDL Isolation and oxLDL Preparation

LDL was isolated and prepared as previously described [7]. LDL was isolated by ultracentrifugation (2 × 24 h, 59,000 rpm at 4 °C) with potassium bromide density adjustments (0.01906 and 0.06583 g/mL KBr/plasma). LDL was dialyzed against PBS (4× against 1 × PBS, pH 7.4 at 4 °C for 1 h, 2 h, 3 h, and overnight) to remove residual potassium bromide. Then, LDL was oxidized using 20 µmol CuSO_4_/L (for 24 h at 37 °C) followed by further dialysis and sterile filtration (pore diameter 0.45 µm). Protein concentrations were measured with Pierce Modified Lowry Protein Assay Kit (ThermoFisher, Rockford, IL, USA, #23,240) according to the manufacturer’s instructions. Endotoxin contamination was tested (<0.01 EU/mL) by ToxinSensor chromogenic LAL endotoxin assay kit (GenScript, Piscataway, NJ, USA, #ABIN491527) according to the manufacturer’s instructions.

### 4.3. Monocyte Priming Experiments

Monocytes were cultured at a density of 30.000 cells/well in a 96-well plate (Greiner Bio-OneTM) and primed with 20 μg/mL oxLDL, 2 μM T0901317 (T1317, LXR agonist, Cayman, Ann Arbor, MI, USA, #71810), 2.5 μM GSK2033 (LXR antagonist, Cayman, Ann Arbor, MI, USA, #25443), 1 μM SR9238 (LXR inverse agonist, Cayman, Ann Arbor, MI, USA, #18771), or vehicle for 24 h in RPMI supplemented with 10% pooled human AB serum, 20 ng/mL MCSF (ImmunoTools GmbH, Friesoythe, Germany, #11343115), 5 mM glucose, and 1% Penicillin/Streptomycin. The inhibitors were added to the culture wells prior to adding oxLDL.

After 24 h the medium was exchanged, and cells were rested for 5 days with 50% of the medium refreshed on day 3. On day 6 the cells were restimulated in a fresh medium (200 µL/well) with 5µg/mL Pam3cys (EMC mirocollection, Tübingen, Germany, #L2000). After 24 h, the supernatant was collected for cytokine assay as well as glucose and lactate assays. In this cell culture model of trained innate immunity neither TLRs nor inflammatory cytokine expression was induced in a relevant manner by the applied doses of oxLDL in human macrophages. The trained immunity phenotype describes modulation of metabolic and epigenetic pathways that pave the way for a stronger inflammatory response to a secondary stimulus. Without this stimulus the inflammatory activity is negligible. However, restimulation with TLR2 agonists induces a proinflammatory response and this reveals the boosting effect of the initial oxLDL training. This observation is not limited to oxLDL priming. Our previous data indicate that the level of cytokine production in response to the training stimulus (LXR agonist) without TLR-restimulation was not measurable [16]. The enhanced cytokine production manifests only after Pam3cys restimulation, as the trained cells show significantly higher cytokine synthesis.

There is a complex interplay between oxLDL and TLR-signaling. In fact, we and others have previously shown that the induction of a trained phenotype through oxLDL stimulation is dependent on TLR-signaling. However, neither TLR nor inflammatory cytokine expression is induced in a relevant manner by the applied doses of oxLDL in human macrophages. With regard to THP-1 cells, for our model, they first need to be differentiated into macrophages. Otherwise, as leukemia cells, the cells continue to divide, which alters the experimental setup. Neither PMA treatment, which induces differentiation, nor oxLDL treatment of PMA-differentiated THP-1 cells resulted in a measurable response of inflammatory cytokines. This is particularly true for the time points analyzed in our study.

### 4.4. Cytokine Measurements

The DuoSet ELISA kits were utilized according to the manufacturer’s instructions to measure the concentration of the pro-inflammatory cytokines: human TNFα (R&D, Minneapolis, MN, USA, #DY210) and human IL-6 (R&D, Minneapolis, MN, USA, #DY206) in cell culture supernatants. The quantified absorbance was determined by the multimode plate reader Victor X3, P Perkin Elmer (Waltham, MA, USA) at 450 nm, and the concentrations were calculated by four parameters of logistic regression.

### 4.5. RNA Isolation and qPCR

Monocytes were cultured with the density of 0.5 and 3 million cells for RNA isolation and harvested 24 h or 6 days after priming respectively. RNA was obtained from the lysed monocytes using the NucleoSpin RNA-isolation kit (Macherey-Nagel, Düren, Germany) and reverse-transcribed using the RevertAid First Strand cDNA Synthesis Kit (Thermo Scientific, Vilnius, Lithuania) according to the manufacturer’s instructions. Real-time qPCR was conducted with the iTaqTM Universal SYBR Green supermix (Bio-Rad, Hercules, CA, USA, #172-5124) to determine the level of expression for *GLUT1*, *HK2*, *PFKFB3*, *LXRα*, *LXRβ*, *ABCA1*, *ABCG1*, *SREBP1*, *SREBP2*, *FASN*, *ACLY,* and *TFIIB* as a housekeeping gene (Table 1).

### 4.6. Chromatin Immunoprecipitation Assay

Monocytes were cultured in a 6-well plate and primed as described before. The cells were fixed, cross-linked, and harvested on day 6 for chromatin immunoprecipitation (ChIP). The assay was performed using the MAGnify Chromatin Immunoprecipitation System (Invitrogen, Carlsbad, CA, USA) according to the manufacturer’s instructions. The cells chromatin was immunoprecipitated with the H3k4me3 (Diagenode, Ougréé, Belgium, #C15410003) or H3K27ac (Diagenode, Ougréé, Belgium, #C15410196) antibody. The rabbit polyclonal IgG (Diagenode, Ougréé, Belgium, #C15410206) antibody was utilized as a negative control. Real-time qPCR amplified the immunoprecipitated DNA using SYBR green and histone enrichment was identified with primers for the *IL-6* and *TNFα* promoters (Table 1). No PCR signal was detected for the IgG control.

### 4.7. Lactate Assay

Monocytes were cultured in 6-well plates and were treated as described above. On day 6 intracellular lactate concentration was measured via colorimetric L-Lactate assay kit according to the manufacturer’s instructions (Abcam, Cambridge, UK, #ab65330): the cells were washed with ice-cold PBS, scraped from the bottom of the plate, and lysed with assay buffer. Cell lysates were deproteinized by spinning through a 10 kD Spin column (Abcam, Cambridge, UK, #ab93349) and thus, eliminating endogenous LDH. Absorbance was measured at 570 nm with the CLARIOstar Microplate Reader and the level of lactate was deduced.

### 4.8. Glucose Consumption Assay

Monocytes were cultured in a 96-well plate and treated as described above. Supernatants were collected on day 6 for glucose concentration measurement using a colorimetric glucose assay kit by Abcam (Cambridge, UK, #ab65333) according to the manufacturer’s instructions. Absorbance was measured at 570 nm with a CLARIOstar Microplate Reader and glucose consumption was calculated.

### 4.9. Acetyl-Coenzyme A Assay

Changes in acetyl Co-A concentration were assessed in cell lysate using a colorimetric commercially available kit (Sigma, St Louis, MO, USA, #MAK039) and performed according to the manufacturer’s instructions.

### 4.10. THP1 Cell Culture, Differentiation, and Priming

In 96 well plates (100 μL/well), 0.35. million cells/mL of the human monocytic cell line THP-1 were cultured, 12 well plates (1 mL/well) or 6 well plates (2 mL/well) in RPMI-1640 medium (Gibco; Paisley, UK) supplemented with 5 mM glucose, 10% FBS (Sigma, St Louis, MO, USA, #F7524) and 1% penicillin/streptomycin (Gibco, Paisley, UK, # 15140122). To induce differentiation, the cells were treated with 100 ng/mL phorbol-12 myristate-13 acetate (PMA) for 24 h. Induction of differentiation is necessary, as THP-1 cells would otherwise continue proliferation, which alters the experimental setup. The cells were washed with warm PBS and were allowed to differentiate for 72 h in a fresh complete medium. The medium was changed and the cells were primed with 20 μg/mL of oxLDL. The cells were rested for 4 days and restimulated with 10 μg/mL of Pam3cys for 4 h or 24 h for RNA isolation or cytokine measurement, respectively. RNA samples were isolated for qPCR analysis and supernatants were collected for ELISA cytokine assay as described earlier. Similar to oxLDL priming in primary human cells, neither PMA treatment nor oxLDL treatment of PMA-differentiated THP-1 cells resulted in a measurable response of inflammatory cytokines.

### 4.11. THP-1 Cell Transfection

Differentiated THP-1 cells were transfected with 30 nM small interfering RNA (siRNA) for *LXRα* (Santa Cruz, Dallas, TX, USA, #sc- 38828), *LXRβ* (Santa Cruz, #sc-45316), *SREBP1* (Santa Cruz, Dallas, TX, USA, #sc-36557), *SREBP2* (Santa Cruz, Dallas, TX, USA, #sc-36559) or Scramble RNA (Santa Cruz, Dallas, TX, USA, #sc-37007) using the Lipofectamine RNAiMAX Transfection Reagent according to the manufacturer’s instructions (Thermofisher, Carlsbad, CA, USA, #13778075). The knockdown efficiency was confirmed by qPCR, 24 h post-transfection (Figure S5A–C). The transfected cells either remained untreated or were treated with 20 µg/mL oxLDL for 24 has described earlier. The cells were rested for 4 days and restimulated with 10 µg/mL of Pam3cys. Additionally, the concentrations of IL-6 and TNFα in the supernatant were measured using ELISA.

### 4.12. Western Blotting

Protein expression of LXRα and PFKFB3 was estimated using Western blotting, with 0.7 × 10^6^ THP1 cells seeded in a 6-well cell culture plate. The cells were differentiated with PMA as it was described earlier. Differentiated cells were treated with 20 μg/mL oxLDL, 2.5 μM GSK2033, or vehicle for 24 h and were rested for 4 days. The cells were harvested for Western blotting using RIPA buffer containing 1% phosphatase (Thermo Scientific, #78426) and 1% protease (Thermo Scientific, Rockford, IL, USA, #87786) inhibitors. Equal amounts of proteins were loaded on 10% SDS-PAGE. Proteins were transferred on a PVDF membrane (GE Healthcare, Uppsala, Sweden, #10600023) and blocked in 5% milk (*w*/*v*) in Tris-buffered saline supplemented with Tween 20 (TBS-T). Membranes were incubated with a primary antibody LXRα (Life Technologies, Rockford, IL, USA, #PA1330; 1:1000 dilution), PFKFB3 (Cell Signaling, Danvers, MA, USA, #13123S; 1:1000 dilution) and GAPDH Antibody (Proteintech, St Leon-Rot, Germany, #60004-1; 1:1000 dilution) in TBS-T overnight at 4 °C, followed by washing with TBST and incubation with a secondary antibody (Goat anti-rb IgG-HRP, Santa Cruz, Dallas, TX, USA, #sc-2004; 1:10000 dilution or Goat anti-mouse IgG-HRP, Santa Cruz, Dallas, TX, USA, #sc-2005; 1:10000 dilution) in TBS-T for 1 h. The blots were washed as described above, developed using Pierce Western blotting substrate (Thermo Scientific, Rockford, IL, USA, #32106), and images were captured using an Amersham Imager 600 (GE Healthcare, Uppsala, Sweden). The intensity of bands was quantified and normalized using ImageJ software.

### 4.13. Statistical Analysis

The differences among experimental groups were evaluated with one-way ANOVA followed by Tukey’s multiple comparisons test using Graph Pad Prism for Windows. The data were checked for normality before analyzing with ANOVA. An F-test was run to check the homogeneity of variance before performing the analysis. The exact number of individual samples and different experiments is mentioned in the Figure legends. Technical replicates were averaged to limit the impact of experimental variations. A *p*-value of < 0.05 was considered statistically significant.

## 5. Conclusions

In conclusion, the cell culture model of oxLDL-induced trained immunity is selectively induced via LXRα. LXR antagonist exerts its effects by blocking the metabolic modulation associated with trained immunity including the upregulation of genes involved in glycolysis and fatty acid synthesis. In addition, LXR inhibition leads to the blocking of epigenetic reprogramming and decreased synthesis of inflammatory cytokines in response to TLR-restimulation.

## Figures and Tables

**Table 1:** Primers used for the qRT-PCR analysis of mRNA expression and immunoprecipitated chromatin.

qRT-PCR Primers for Gene Expression Analysis		
Gene	Forward (5′ to 3′)	Reverse (5′ to 3′)
GLUT1	CGGGCCAAGAGTGTGCTAAA	TGACGATACCGGAGCCAATG
HK2	TTGACCAGGAGATTGACATGGG	CAACCGCATCAGGACCTCA
PFKFB3	ATTGCGGTTTTCGATGCCAC	GCCACAACTGTAGGGTCGT
LXRa	GTTATAACCGGGAAGACTTTGCCA	GCCTCTCTACCTGGAGCTGGT
LXRb	CGTGGACTTCGCTAAGCAAGTG	GGTGGAAGTCGTCCTTGCTGTAGG
ABCA1	ACCCACCCTATGAACAACATGA	GAGTCGGGTAACGGAAACAGG
ABCG1	ATTCAGGGACCTTTCCTATTCGG	CTCACCACTATTGAACTTCCCG
SREBP2	AGGAGAACATGGTGCTGA	TAAAGGAGAGGCACAGGA
SREBP1	GCAAGGCCATCGACTACATT	GGTCAGTGTGTCCTCCACCT
FASN	CAGGCACACACGATGGAC	CGGAGTGAATCTGGGTTGAT
ACLY	AACCCCAAAGGGAGGATCT	TTGACACCCCCTAGATCACAG
h-TFIIB	TCGCCACATTCGCTTCCTGCTTTC	ATATCACCGGCTCTGTAGTCCTCCAC
qRT-PCR primers for analysis of immunoprecipitated chromatin		
Region	Forward (5′ to 3′)	Reverse (5′ to 3′)
TNF promoter	ACTTTCCAAATCCCCGCCCCC	GTGTGCCAACAACTGCCTTTATATGTCC
IL6 promoter	AGGGAGAGGGAGCGATAAACACAAAC	TTCACTGGGGCACCTGCATGG

## Data Availability

Not applicable.

## Supplementary Materials

The following supporting information can be downloaded at: https://www.mdpi.com/article/10.3390/ijms23116166/s1.
