# Supplementary material for: LXRα Regulates oxLDL-Induced Trained Immunity in Macrophages
Source: Int J Mol Sci. 2022 May 31;23(11):6166. doi: 10.3390/ijms23116166 (PMC9181299; doi:10.3390/ijms23116166)
Supplement: Supplementary file 1 [file ijms-23-06166-s001.zip › ijms-1644314-supplementary.pdf]

International Journal of *Molecular Sciences*

MDPI

LXRα Regulates oxLDL-Induced Trained Immunity in Macrophages

Hannes M. Findeisen [1#*], Vivienne C. Voges [#1], Laura C. Braun [1], Jannik Sonnenberg [1], Dennis Schwarz [1], Helena Körner [1], Holger Reinecke [1] and Yahya Sohrabi [1*]

Supplementary Figures:

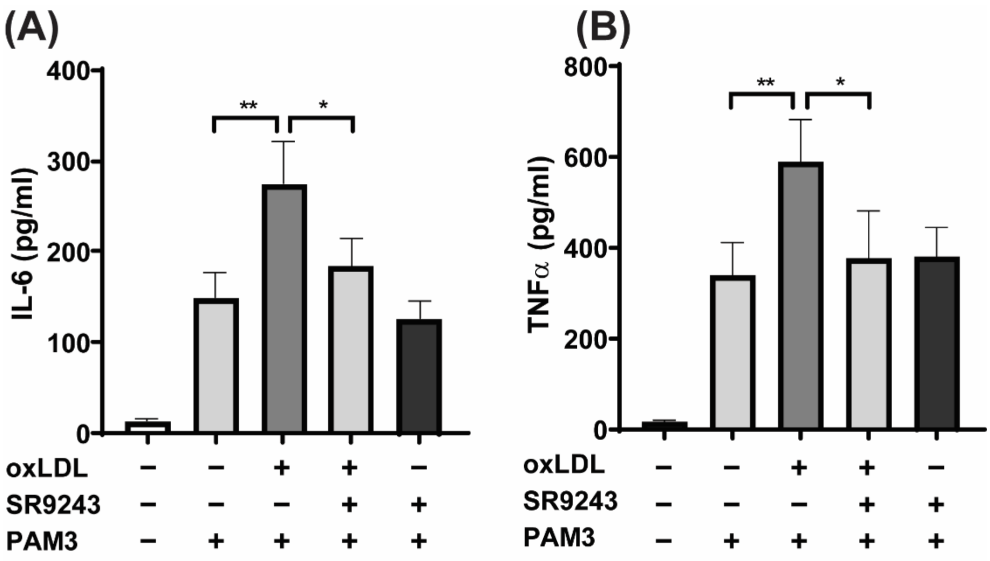

Figure S1. LXR inverse agonist inhibits oxLDL-induced proinflammatory immune memory in human monocytes. Monocytes were treated as indicated with 20 µg/ml oxLDL, 1 µM SR9238 (LXR inverse agonist) or vehicle for 24 h, kept for 5 days in complete medium and restimulated with 5 µg/ml Pam3cys for 24 h. IL-6 (A) and TNFα (B) were measured in the supernatant using ELISA. Graphs represent mean values ± SD of six individuals in three different experiments. *$P < 0.05$, **$P < 0.01$.

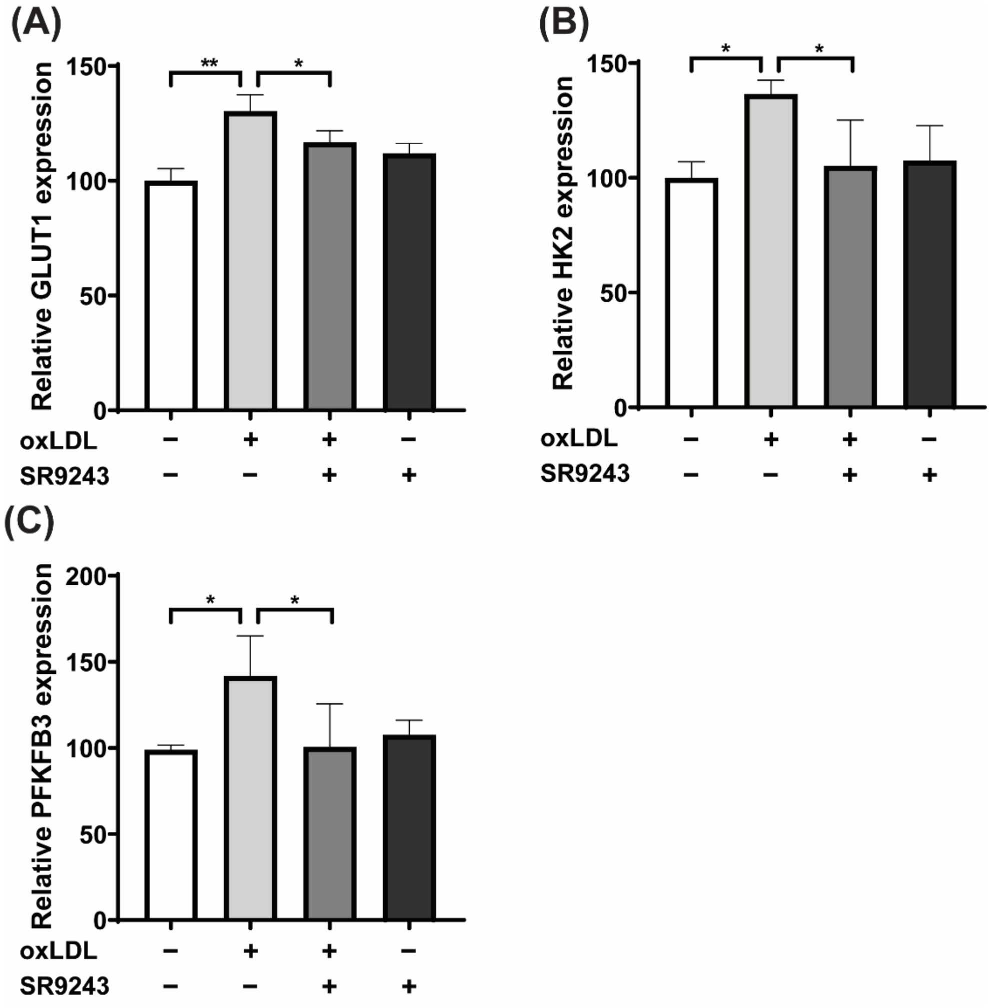

Figure S2. LXR inhibition alters oxLDL-induced metabolic reprogramming in trained monocytes. Monocytes were treated as indicated with 20 µg/ml oxLDL, 1 µM SR9238 (LXR inverse agonist) or vehicle for 24 h and lysed for RNA expression. mRNA levels of *GLUT1*, *HK2* and *PFKFB3* were analyzed by real-time qPCR (A–C). Graphs represent mean values ± SD of six individuals in three different experiments. *$P < 0.05$ and **$P < 0.01$.

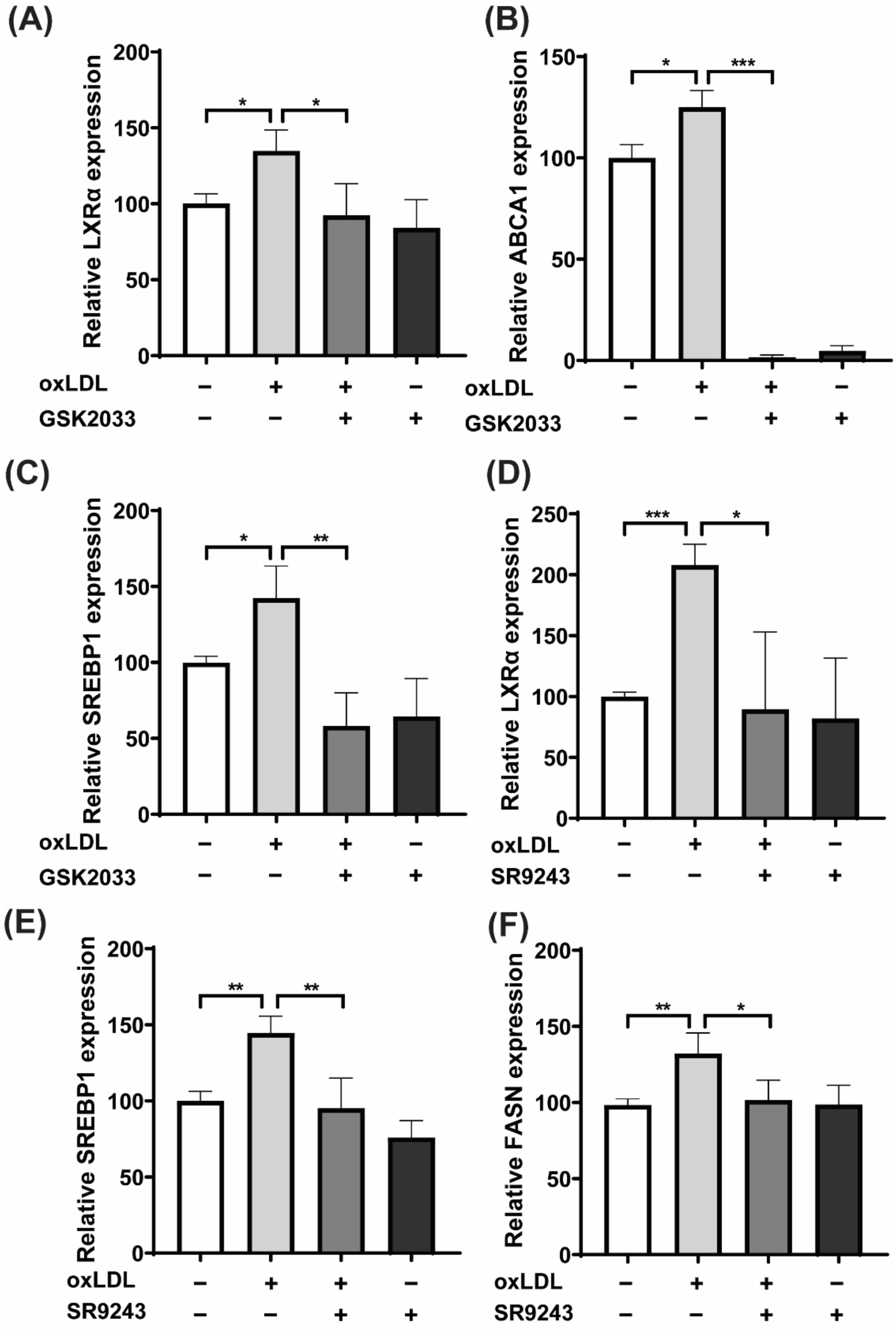

Figure S3. LXR inhibition reduces oxLDL-induced upregulation of LXR target genes. A–C: THP1 cells were treated with 20 μg/ml oxLDL, 2.5 μM GSK2033 (LXR antagonist) or vehicle for 24h and lysed for mRNA expression. mRNA expression of *LXRα*, *ABCA1*, *SREBP1* was measured using qPCR. D–F: Human monocytes were treated with 20μg/ml oxLDL, 1 μM SR9238 (LXR inverse agonist) or vehicle for 24 h and lysed for gene expression analysis. Expression of *LXRα*, *SREBP1* and *FASN* genes were analyzed by qPCR. Graphs represent mean values ± SD of six individuals in three different experiments. *$P < 0.05$, **$P < 0.01$, and ***$P < 0.001$.

International Journal of
Molecular Sciences

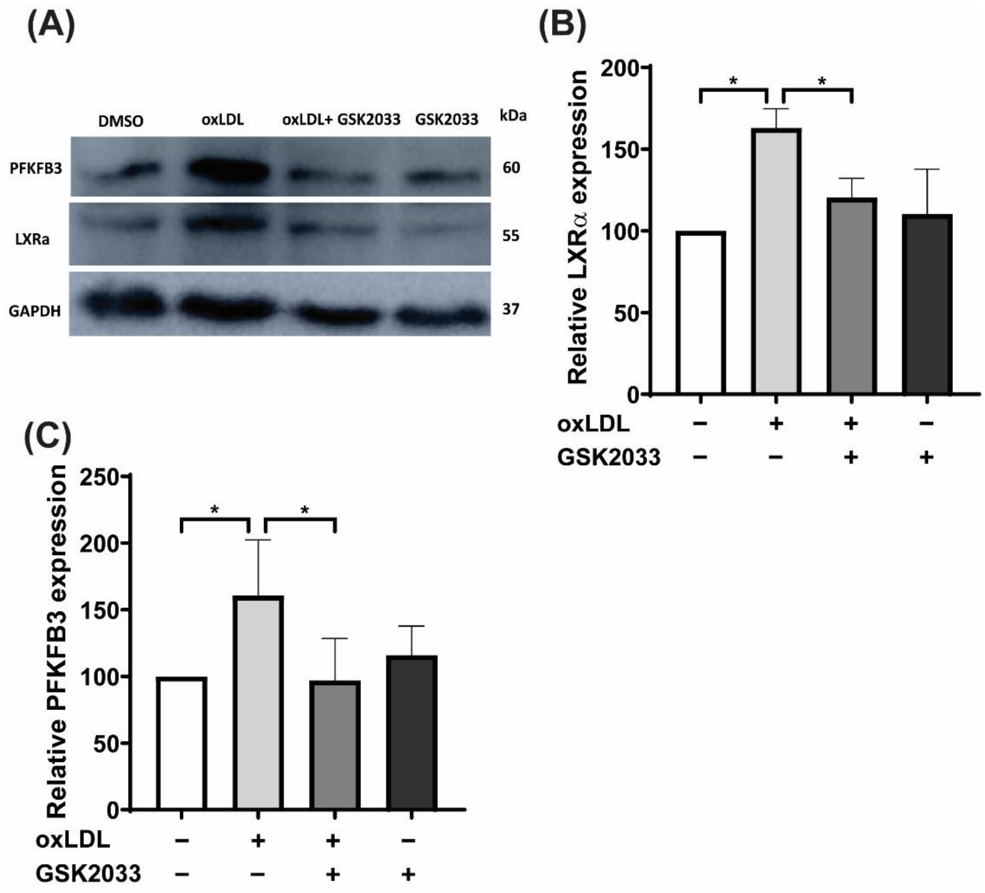

Figure S4. LXR antagonist inhibits oxLDL-induced upregulation of LXRα and PFKFB3 protein concentration in trained THP1 cells. Differentiated THP1 cells were treated with 20 μg/ml oxLDL, 2.5 μM GSK2033 (LXR antagonist) or vehicle for 24h. The cells were rested for 4 days and whole-cell extracts were subjected to western blot analysis for LXRα and PFKFB3 protein analysis. (A–C). Graphs represent mean values ± SD of six individuals in three different experiments. *$P < 0.05$.

International Journal of
Molecular Sciences

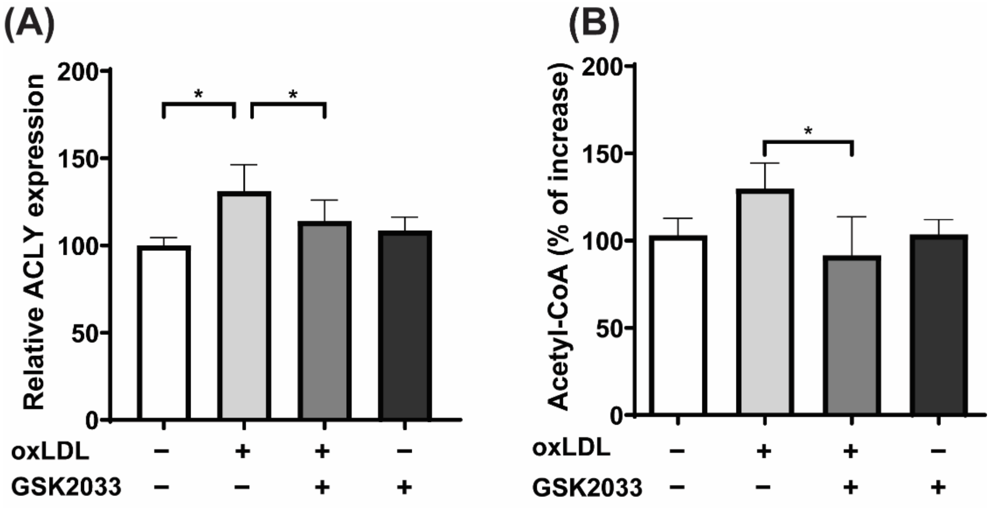

Figure S5. LXR inhibition alters oxLDL-induced Acetyl-CoA production. Monocytes were treated as indicated with 20 μg/ml oxLDL, 2.5 μM GSK2033 (LXR antagonist) or vehicle for 24 h and either lysed for RNA expression or rested for 3 days in complete medium. mRNA level of *ACLY* gene was analyzed by real-time qPCR (A). Cells were lysed and Acetyl-CoA concentration was measured on day 3 (B). Graphs represent mean values ± SD of six individuals in three different experiments. **P* < 0.05.

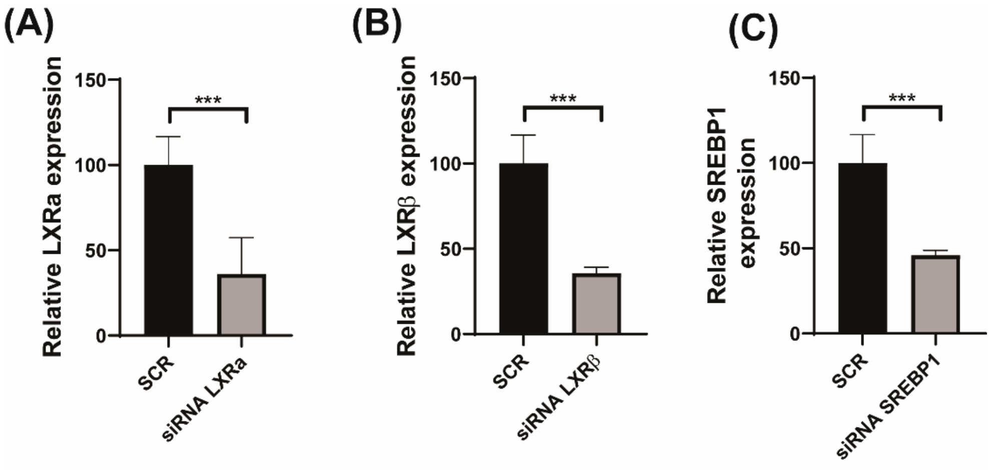

Figure S6. siRNA transfection efficiency. Differentiated THP1 cells were transfected with siRNA against *LXRα*, *LXRβ* and *SREBP1* or scrambled siRNA for 24h and lysed to check transfection efficiency. mRNA expression of *LXRα*, *LXRβ* and *SREBP1* was measured using qPCR (A–C). Graphs represent mean values ± SD of six individuals in three different experiments. ****P* < 0.001.
